# Using collaborative approaches with a multi-method, multi-site, multi-target intervention: evaluating the National Research Mentoring Network

**DOI:** 10.1186/s12919-017-0085-6

**Published:** 2017-12-04

**Authors:** Lourdes R. Guerrero, Jennifer Ho, Christina Christie, Eileen Harwood, Christine Pfund, Teresa Seeman, Heather McCreath, Steven P. Wallace

**Affiliations:** 1 Division of General Internal Medicine/Health Services Research, David Geffen School of Medicine at the University of California, Los Angeles, CA USA; 2 Department of Education, Graduate School of Education and Information Sciences, University of California, Los Angeles, CA USA; 3 Division of Epidemiology and Community Health, University of Minnesota - Twin Cities, Minneapolis, MN USA; 4 Department of Medicine and Wisconsin Center for Education Research, University of Wisconsin-Madison, Madison, WI USA; 5 Division of Geriatrics, David Geffen School of Medicine at the University of California, Los Angeles, CA USA; 6 Department of Community Health Sciences, UCLA Fielding School of Public Health, Los Angeles, CA USA

## Abstract

**Background and purpose:**

The NIH-funded National Research Mentoring Network (NRMN) aims to increase the representation and success of underrepresented groups (URGs) in biomedical research by enhancing the training and career development of individuals from diverse backgrounds, communities, and cultures. The national scope of NRMN, its wide array of innovative programs in mentor and mentee matching and training across the career spectrum, requires a collaborative evaluation strategy that addresses both internal and external evaluation needs. Due to the variety of programs implemented for each target group, the NRMN program is responsible for its own process and short-term outcome evaluations and the national Coordination and Evaluation Center (CEC) is responsible for assessing the medium and long-term effectiveness of the implemented strategies and program sustainability. Using a collaborative, utilization-focused evaluation framework, both internal NRMN evaluators and the CEC are working to translate findings into information that can be used to make both short term and long-term decisions about the efficacy and reach of the NRMN model. This important information can then inform efforts to institutionalize the current programs and potentially replicate them elsewhere.

**Program and key highlights:**

The overall evaluation of NRMN is guided by both outcome and process questions that are tailored for each target group. The different target groups include faculty and others who serve as mentors, mentees across academic training and career stages, and researchers without a history of independently funded research. NRMN is also building the capacity for training those pursuing biomedical careers by developing “master trainers” for both mentoring and grantsmanship programs in organizations and institutions that can support expanded training efforts aimed at diversifying the biomedical workforce.

**Implications:**

Results of this evaluation will be used to inform the design and implementation of sustainable, effective, and comprehensive mentoring and career development initiatives that promote diversity in the biomedical research workforce. Our collaborative evaluation design, theoretically-derived measurement instruments, efficient data systems, and timely reporting serve as an example of how to put evaluation principles described into practice for large, multi-site, and multi-dimensional research training programs like NRMN.

## Background

The overall goal of the National Research Mentoring Network (NRMN) is to increase the diversity of the biomedical research by enhancing mentorship and career development of individuals from diverse backgrounds, communities, and cultures. The NRMN implements a complex set of interventions and delivery mechanisms to reach trainees, educators and researchers in biomedical disciplines across the United States to improve their skills as mentors and mentees, and their ability to be effective grant proposal writers. The long-term goal is to increase the number of individuals from underrepresented groups (URGs) who become NIH funded researchers [1]. We have adopted the URG terminology used by NRMN since the NIH priorities for diversifying the research workforce include individuals from underrepresented racial/ethnic minorities, persons with disabilities, and individuals from other disadvantaged backgrounds, such as low-income families [2]. Evaluating the efficacy and reach of NRMN programing in the short and long term is critical as it informs which interventions NIH and other research organizations can use in their efforts to diversify of the research workforce.

The innovative format, complexity of activities, and national scope of NRMN activities require a coordinated evaluation to determine the effectiveness of strategies and sustainability of the efforts. This evaluation is especially important given the limited empirical evidence on the *value* of mentoring programs specifically in higher education and the challenge of measuring theory-informed short-term outcomes [3]. This article describes the early-stage development and implementation of the collaborative evaluation plan for the NRMN, the results of which will be critical to understanding how to improve mentoring and professional development for URGs, and, in turn, support their sustained biomedical research career success.

The National Institutes of Health Diversity Program Consortium (NIH DPC) is the national collaborative under which the NRMN, as well as the Coordination and Evaluation Center (CEC) at UCLA, were established. The overall Consortium-Wide Evaluation Plan (CWEP) Hallmarks of Success (both short and longer-term outcomes of the project) [4], and NRMN and CEC logic models were used to guide the evaluation plan for NRMN. NRMN program evaluators and CEC evaluators developed this plan collaboratively over time. NRMN principal investigators and their internal evaluation team worked together with the CEC to ensure that the CEC plan and its implementation reflected the interests and focus of both the NRMN and CEC, covered both shorter-term and longer-term outcomes, and that relevant evaluation activities were incorporated into the broad Consortium-wide evaluation plan. The nationwide scope of NRMN, its organization into multiple cores with diverse activities, and the high level of research expertise on specific activities in each NRMN core led the consortium to design the external evaluation as a sequential process rather than a more traditional overlapping process. That is to say, conventionally an external evaluator would examine a project simultaneously with any internal program evaluation, with each having different foci. The CEC’s evaluation of NRMN, on the other hand, is tightly coordinated over time. The CEC has worked with NRMN to assure that their short-term evaluation metrics include participant data needed for longer-term follow-up and at the completion of their program the NRMN hands off participants’ baseline data to the CEC for the longer-term component of the evaluation.

The evaluation of the NRMN outcomes is guided by both process and short-term outcome questions. Representative short-term questions to be answered by NRMN evaluators include: What strategies are used to promote the value of mentoring and the NRM network nationwide? What immediate and short-term changes in participants result from exposure to NRMN programs (e.g. access to new mentors, improved mentoring relationships, improved grantsmanship skills, increased self-efficacy, etc.)? Does the reach of NRMN meet, exceed, or fall short of delivering outcomes necessary to make significant contributions to the broader, long-term NRMN goal of diversifying the biomedical workforce?

Longer-term questions to be answered by CEC evaluators include: What is the effect of NRMN participation on medium-term predictors of long-term success as NIH-funded researchers (e.g. grant submissions, published research articles, career advancement), especially for those from URGs? Does the effect of NRMN programs on participants (e.g., mentoring and grant writing skills) continue over time? How well do the various programmatic components of NRMN interact with each other and how does this impact program capacity, success, and sustainability? These evaluation questions were drawn from an integrated logic model developed by NRMN (Table 1) and the relevant DPC Hallmarks of Success, as well as the required outcomes in the NIH Request for Application.

**Table 1:** NRMN Integrated Logic Model

Program Components	Goals	Key Program Outputs	Key Short-term Outcomes
Match/link mentees to mentors and coaches	Increase access to mentoring across all career stages	Number of mentors and mentees engaged in mentoring relationships Number of mentors, mentees, coaches matched through NRMN Number of mentors and mentees engaged in MyNRMN	Increased number and diversity of individuals engaged in mentoring relationships Increased size of mentoring networks for individuals and groups
Train mentors, coaches & mentees	Train and certify mentors Train grant writing coaches Train mentees Improve cultural responsiveness in mentorships	Number individuals recruited, trained Number individuals certified Number of evidence-based, career-stage-tailored training programs	Increased # of trained mentors and coaches available to work with individuals at all career stages in the biomedical research field Increased quality of culturally-sensitive mentoring and coaching of individuals across career stages at an increased number of US colleges and universities
Refer mentees to career and research resources	Increase access to research resources and career development opportunities	Number of organizations partnering with NRMN to provide career and research resources to NRMN trainee and mentee participants Number of resources offered through and in partnerships with NRMN Number of individuals using resources	Increased access to career-enhancing resources, offered through and beyond NRMN offerings, by a diverse population of individuals and higher education institutions Increased size and quality of a network of organizations, associations, and qualified mentors and researchers committed to ensuring long-term diversity in the biomedical workforce
Promote the value of career mentoring across the nation	Partner with professional societies and organizations Increase awareness at all levels: individuals, institutions, associations	Number professional societies and organizations partnering with NRMN to promote career mentoring Number of entities promoting mentoring and NRMN	Increased recognition at colleges/universities of the value of mentoring for diverse workforce at all career stages Increased national level recognition of efforts to diversify the US biomedical workforce achieved through NRMN career and grant writing mentoring and coaching

### Evaluation approach

The NRMN evaluation is characterized by its utilization-focused approach [5, 6], fulfilling one of the CEC’s fundamental roles in turning data into information that can be used to inform decisions across the Diversity Program Consortium, as well as being an important source of information for NRMN program leadership and NIH (i.e., decision makers) [7]. This approach offers a set of principles for conducting evaluations that support data-based decisions and promote individual and organizational learning. To foster meaningful use of data within NRMN, a concerted effort is made to engage users of evaluation data and findings.

To facilitate organizational learning, NRMN established an evaluation team, which is comprised of approximately 12 to 15 representative members from across the NRMN network plus members of the CEC evaluation team. This team is highly collaborative and brings together a community of researchers who listen, contribute to, critique, and advance the evaluation work including developing strategies to promote use of the evaluation and its findings.

To support active use of information by program leaders and implementers, the evaluation is focused on addressing questions related to mentor and mentee skills, research self-efficacy, trainee experiences and early-career research outcomes. A common set of shared measures was identified for use by all program components so that data are used to inform both the short and long term evaluation questions. These shared measures are compiled in an NRMN Measurement Library developed by members of the NRMN Evaluation Team. To promote use, performance metrics are not only reported and/or made available to all stakeholders (i.e., NRMN program leaders, CEC evaluation team members and the DPC Executive Steering Committee), but are the basis for evaluation project data gathering and analysis discussions. Presentations and discussions regarding both the short term and long term evaluation activities and emerging findings are embedded within annual consortium-wide meetings, biannual NRMN Key Personnel Meetings, and bimonthly NRMN Evaluation Team meetings, as well as a variety of program-level meetings that occur across NRMN’s national network. NIH program representatives participate in many of these meetings and events, providing continuous input concerning performance metrics that are important to NIH stakeholders. The NRMN and CEC receive funding through a cooperative agreement with NIH, which provides much greater sponsor involvement in program implementation and evaluation than in traditional research or training grants. This coordinated effort assures that measured and tracked evidence and outcomes meet the needs of program administrators, as well as external stakeholders including NIH.

Alongside a collaborative, utilization-focused approach, the national NRMN evaluation intentionally looks at context for the evaluation. In this way, the NRMN evaluation recognizes the central role of contextually-defined values and beliefs held by program stakeholders and their program evaluators [8]. By paying attention to the context in which the evaluation will be conducted (for example, in asking what are the stories of communities and people participating in NRMN, and who is telling them), the NRMN evaluation is designed to yield an accurate, valid, and grounded understanding of what is and who are being evaluated [8]. This contextually-responsive position, coupled with the utilization focused evaluation approach complement overall Consortium goals, ensuring that evaluation stakeholders and participants see themselves as appropriately reflected within the evaluation and central as the ultimate users of its findings.

### Description of the collaborative evaluation design and methods

The NRMN evaluation employs a multi-and mixed methods process and outcome design that includes standard assessment surveys, observations, quasi-experimental, and longitudinal methods. Basic participant demographics and background information include information collected through the NRMNet portal and registration system. Administrative and process outputs, and participant short-term outcome measures are tracked by NRMN program staff and/or evaluators across NRMN in a variety of data systems created during the program’s start-up stage. These measures cover some of the following outputs for programs and participants: program created (descriptions) and events offered (types, description, location, start/end dates, expected/actual enrollment counts, and, for many, participant rosters); pre−/post-intervention metrics related to satisfaction, skills, self-efficacy, planned or actual self-reported changes in participant behavior (where applicable), and, for early career researchers in the grant writing programs, measures of change over time over up to 18 months post training to identify grants written, submitted, revised, abandoned, awarded, and resulting research publications within that timeframe.

Longer-term outcomes that are included among the Hallmarks of Success will be tracked over time by the CEC. Hallmarks that apply to NRMN include increased research productivity (as measured by research grant submissions and peer-reviewed publications of early-career faculty and post-doctoral trainees), the improved and sustained perceptions of self-efficacy of trained mentors and early career researchers, and the increased availability of trained mentors for mentees across the country. Additionally, several longer-term outcomes address NRMN’s impact on institutionalization including indicators of program sustainability and the formation of durable networks and partnerships. These data will initially be collected in conjunction with the NRMN’s process evaluation and then the CEC will continue to collect relevant data for long-term outcomes.

#### Sample

NRMN’s primary targets are early career professionals (post-doctoral and junior faculty) as well as postsecondary students (undergraduate through doctoral) who are training for, or are engaged in, biomedical research studies or careers, as well as those mentoring these students and early career researchers. Secondary targets are those who can serve as trainers and coaches to the primary targets. Hence, NRMN’s primary participants engage in activities as either trainees / mentees or mentors, or both, and NRMN secondary participants are those who train or coach the primary participants.

*Mentorship* has been defined as a reciprocal, dynamic relationships, between mentors and mentees that promote the satisfaction and/or development of both [9]. A mentor has also been defined as a person who is assigned to work with you on research or who is responsible for providing direction to you, supervising you, helping you, answering your questions, etc. For the CEC, a mentor is someone who provides guidance, assistance, and encouragement on professional and academic issues. For NRMN, mentors are also those advising or coaching mentees in grant writing development and/or career planning. Mentors and coaches can be engaged in a variety of relationships with mentees, including as peers, informally and informally-linked and formally defined relationships. The CEC defines mentees as someone who receives guidance or assistance from a mentor. NRMN defines *mentees* as those seeking and receiving the “(1) personal and professional competencies necessary to define their career goals, (2) experience needed for realizing their career goals, and (3) ability and opportunity to progress toward their chosen career goal” [10]. The CEC and NRMN use slightly different definitions for mentors and mentees for accountability and reporting purposes, as the NRMN short-term evaluation needs to account for participation of individuals in designated roles and the CEC evaluation is more focused on the long-term effects of the programs.

NRMN participants are identified from those who register on the NRMNet web portal, which is the common registration site for all NRMN programs. Registrant lists include contact information and basic demographics for the wide range of persons interested in NRMN and its events and activities. Where it is gathered, baseline and immediate post-program assessments for event participants is provided by NRMN for CEC’s follow-up analysis. As part of the collaborative process, some of the individual programs modified their initial data collection protocols to enable the CEC to conduct an individual-level longitudinal follow-up.

The CEC will maintain a sample of program participants who are primary targets, with the periodic addition of new registrants. The evaluation challenge is to identify similar persons matched across different types of institutions nationally who did not participate in the NRMN programs. In educational programs, in particular, it is important to distinguish changes in skills and career standing that follow an intervention from changes that would have occurred without the intervention. To best construct a quasi-experimental control group for the self-selected, heterogeneous participants in NRMN programs, we sample others who registered on the NRMN web portal whose career stage and discipline are similar but who, for whatever reason, did not participate in the target NRMN programs and events (see Sorkness et al., this volume for a full description of NRMN activities). This group is likely to have a similar level of interest in biomedical career development as NRMN participants, but were unable to participate or discouraged from participating in the trainings. Along with those who only participated in one NRMN program or event (e.g. early career researchers who completed mentor training but not grantsmanship training), this approach yields several samples of *unexposed* individuals from a range of institutions that can be compared to individuals who engaged in multiple specific NRMN activities.

In addition, the CEC has also innovated by asking the NRMN respondents to nominate two of their mentees for a brief follow-up on mentoring. These nominees are kept in a separate file and retained only if they agree to participate in a survey, at which point they become part of a mentor-mentee dyad for tracking over time. This pairing allows the CEC to track career trajectories of mentees with NRMN trained versus those with similar mentors without NRMN training to identify the value added by NRMN versus other training versus no mentor training.

#### Measures

The CEC and NRMN identified short and long-term outcomes, indicators and measures critical to the evaluation of network activities, and observable changes in participant experiences with mentoring, such as improved access to mentoring relationships, increased confidence in pursuing biomedical careers and in preparing and submitting grant applications. Measures were based on theories, evidence-based models, and validated instruments to explain and describe biomedical research career persistence across diverse groups with an emphasis on measures most likely to be affected by the interventions, such as satisfaction with mentoring, increased science identity, higher self-efficacy and enhanced research productivity (e.g. published articles, grants). A measurement library, developed and maintained by the NRMN evaluation team for use across NRMN activities, is being used to facilitate the use of common, evidence-based metrics across the program [11]. To collaboratively guide the evaluation process, NRMN activities and outcomes were mapped to Consortium Wide Evaluation Plan (CWEP) outcomes as a way to refine its own articulated goals, identify key performance and outcome metrics, and prioritize data collection and analysis activities. Table 2 illustrates the alignment of a sample of outcomes from a list of NRMN Outputs and Outcomes with CWEP Outcomes. While they differ somewhat in scope and emphasis, the elements of the long-term evaluation of NRMN (and other Consortium-wide activities) build on the short-term evaluation of NRMN activities.

**Table 2:** Sample of Integrated NRMN Outputs, Outcomes and CWEP Outcomes

NRMN Outputs and Short-term Outcomes^a^	Medium & Long-term, CWEP Outcomes^b^
Number and types (gender, race & ethnicity, career stage) of mentors and mentees participating in NRMN activities, accessing resources and/or engaging in mentoring relationships through NRMN	Increased participation in mentoring activities (faculty^c^) Increased participation in mentoring activities by URGs (trainees^d^)
Increased mentee self-efficacy in research tasks and addressing difficult situations in research settings	Psychosocial variables including academic and scientific self-efficacy
Increased commitment to cultural change in promoting diversity in biomedical research; increased intention to change institutional culture, policies, structures, and/or activities related to mentoring access	Increased institutional commitment to sustaining activities
Increased number and percentage of faculty/post-docs satisfied with gains in skills/knowledge/confidence / regarding how to write winning grants	Increased research productivity in grant submissions and awards as PI, multi-PI and/or collaborator
Number and type of professional development opportunities offered to the community through NRMN or in partnership with NRMN	Increase, enhance, and/or develop inter-institutional collaborations to achieve BUILD (and related) outcomes related to research, mentorship, and faculty development

^a^ The NRMN integrated logic model includes additional outcomes not noted here

^b^ Taken from the consortium wide hallmarks/logic model

^c^ Faculty includes anyone in the mentoring role and may include graduate students, post-docs, faculty and other researchers/advisors/ coaches

^d^ Trainees include undergraduates, graduate students, post-docs and junior faculty

#### Data systems to track participation

To facilitate NRMN-wide data collection efforts, NRMN developed a data management system to assemble in one place: information about program delivery, participant tracking, and assessments needed for evaluation. The CEC has a compatible but separate system to receive selected data from NRMN to facilitate long-term follow-up and analysis of participant outcomes. The selected data provided to the CEC, including detailed participant contact information and flags regarding participation in activities, facilitate the creation of longitudinal sample groups and provide a means of monitoring participation in CWEP data collection efforts. Simultaneous development of the systems facilitates this exchange of information.

### Data collection methods

#### NRMN participants

As stated previously, process, output and participant short-term outcome measures are gathered and tracked by NRMN program staff and/or evaluators across NRMN programs. In an effort to gain a longer, broader view of NRMN participant experiences, as well as to gauge the impact of NRMN program participation on the outcomes specified by the CWEP, the CEC conducts an annual follow-up survey of NRMN participants after they leave the internal-NRMN evaluation, which occurs anywhere from immediately after a training to 18 months post-training depending on the activity. The CEC survey collects information on career progress, participation in other professional development activities, research skills and activities, involvement in mentoring relationships and programs, mentor/ mentee competency across a range of skills, and perceptions of self-efficacy. This sequential evaluation (internal NRMN then CEC) strategy reduces respondent burden since the two evaluations do not simultaneously collect information from the same participants.

#### NRMN as an organization

Complementing follow-up survey data from participants, the CEC also collects qualitative data through semi-structured interviews with NRMN leadership, including principal investigators, core leaders, and program management staff (approximately 20 individuals per year), and participant observation of NRMN planning meetings to assess organizational development sustainability. This approach informs the quantitative data collection efforts to understand how NRMN programs implement their vision for advancing URG biomedical research training, how the various parts of NRMN interact and collaborate with one another and other Consortium sites and the perceived value of those interactions, and the extent to which NRMN strategies enhance student, faculty, and institutional engagement in biomedical research training for URGs.

More specifically, interviews focus on details surrounding NRMN implementation as a consortium, the perceived strengths and needs of NRMN participants by program staff, network integration, and sustainability efforts. Semi-structured observations of NRMN Key Personnel Meetings (KPM) will hone in on how these events contribute to overarching NRMN goals, cultivate potential synergies across NRMN and Consortium-wide program activities, and eliminate or reduce barriers to effective collaborations and programmatic needs. Similar observations will be conducted of NRMN activities as a way of documenting network structure, context, and achievements. Combined, interviews and observations will inform a deeper, contextually driven understanding of process and activity integrity.

### Data analysis

#### NRMN participants

Short-term outcome evaluation data are necessarily descriptive in the early stages of NRMN development as a network. For instance, change-over-time metrics in participant improvements in awareness, knowledge, skills, self-efficacy, confidence, and professional advancement are limited to measures collected during participation in the programs. Several of these findings will be published to inform the community of what activities show promise for broader dissemination. These data are shared with the CEC for longitudinal studies as described.

CEC survey findings will first be used to present descriptive statistics of longitudinal participant outcomes. Comparative analyses will then be performed among different groups of NRMN registrants, including assessing differences in specific domain areas, such as mentor-mentee relationships, research productivity, and college/career persistence. To control for maturation and other potential threats to validity, we will compare those who participated in specific trainings with those in the database at similar career levels who did not. For example, we will look at the number of people mentored and the mentor’s self-assessed skills for NMRN-trained mentors compared to NRMNet web portal registrants who do not have NRMN training.

Differences will also be investigated among trainees working with NRMN coaches through the grants writing training programs and trainees not participating in the coaching program. We will conduct comparative analyses between mentees engaged in other NRMN programming, such as the virtual guided mentorship program, with those not participating in those or other NRMN mentorship programs. These analyses will involve multivariable methods that control for other career-success predictors, such as discipline, institutional status of graduate degree, prior research experience/grant/publication record, and current institutional context.

Using annual follow-up surveys, cumulative data will be examined in a longitudinal analysis of trends and patterns (i.e., difference in differences) during the course of the project. For example, if the mentor training stimulates senior faculty to increase their number of mentees, does this effect decay over several years and return to the mean? For those who have been coached in grant writing skills, does early mentored success in funding become a sustained record of funded projects after they leave the program. These analyses will also be examined to determine if there is any interaction effect by URG status. These are key issues in promoting the long-term goal of impacting the diversity of the NIH-funded workforce.

#### NRMN as an organization

Observation and qualitative interview data from NRMN leadership and program staff will be analyzed in 4 cycles [12]. Through pattern coding we will synthesize findings into more meaningful units of analysis [13]. By grouping similarly coded passages together and assessing the groupings for thematic similarity, difference, frequency, sequence and correspondence, the final coding scheme will be established. Finally, through elaborative coding, we will examine the data with an eye toward the Consortium-wide Evaluation Plan. The coding team will write analytic memos throughout the coding process that will both document the process of the emerging analysis and also serve as guideposts for the collaborative process.

## Conclusion

### Distinctive features and implications for the evaluation of mentoring

The evaluation of NMRN is informed by a utilization-focused evaluation approach that, by design, engages several critical stakeholder groups. As a collaborative agreement with NIH, we solicit and receive regular feedback concerning the measures and outcomes used to evidence program achievements and challenges in ways that are useful for NIH decision making. The NRMN evaluation team and the CEC work in concert with NRMN leadership to provide continuous feedback in the formative development of their initiatives as they are implemented – data that will later be used to summatively assess NRMN programming in ways that can guide future development and sustainable replication. To facilitate these collaborative actions, deliberate efforts were made to ensure alignment between NRMN program goals with desired Consortium-wide outcomes, and that intermittent results are regularly reviewed to reinforce utility, accuracy, feasibility, propriety, and appropriateness.

The close collaborative working relationship of the CEC and NRMN evaluation teams will continually produce strong and clear plans for coordinated evaluation processes that efficiently and effectively leverage resources (human, financial, technical), knowledge and expertise - that exists across/between both teams. The CEC’s collection of both quantitative and qualitative data imbue the NRMN evaluation with an essential understanding of the context in which NRMN was initiated and implemented, as well as to ensure the many communities of stakeholders contributing to, and benefiting from its programs are both honored and integral to its findings.

The results of this NRMN evaluation will be used to inform national policy on how to best design and implement comprehensive mentoring and career development initiatives for diverse populations pursuing careers in biomedical research. Understanding the complexity of implementing a nationwide mentoring network, as well as the attributes that most support its targeted participants will contribute substantially to the ways in which we consider the role of mentoring to promote researcher diversity in the biomedical sciences.
